# Potassium indole-3-butyric acid affects rice’s adaptability to salt stress by regulating carbon metabolism, transcription factor genes expression, and biosynthesis of secondary metabolites

**DOI:** 10.3389/fpls.2024.1416936

**Published:** 2024-09-03

**Authors:** Hang Zhou, Fengyan Meng, Wenxin Jiang, Xutong Lu, Rui Zhang, Anqi Huang, Kunlun Wu, Peng Deng, Yaxin Wang, Huimin Zhao, Youwei Du, Jingxin Huo, Xiaole Du, Naijie Feng, Dianfeng Zheng

**Affiliations:** ^1^ College of Coastal Agricultural Sciences, Guangdong Ocean University, Zhanjiang, China; ^2^ School of Tropical Agriculture and Forestry, Hainan University, Haikou, China

**Keywords:** potassium indole-3-butyric acid, rice, transcriptome, metabolome, salt

## Abstract

Soil salinity pollution is increasing worldwide, seriously affecting plant growth and crop production. Existing reports on how potassium indole-3-butyric acid (IBAK) regulates rice salt stress adaptation by affecting rice carbon metabolism, transcription factor (TF) genes expression, and biosynthesis of secondary metabolites still have limitations. In this study, an IBAK solution at 40 mg L^−1^ was sprayed on rice leaves at the seedling stage. The results showed that the IBAK application could promote shoot and root growth, decrease sucrose and fructose content, increase starch content, and enhance acid invertase (AI) and neutral invertase (NI) activity under salt stress, indicating altered carbon allocation. Furthermore, the expression of TF genes belonging to the ethylene responsive factor (ERF), WRKY, and basic helix-loop-helix (bHLH) families was influenced by IBAK. Many key genes (*OsSSIIc*, *OsSHM1*, and *OsPPDKB*) and metabolites (2-oxoglutaric acid, fumaric acid, and succinic acid) were upregulated in the carbon metabolism pathway. In addition, this study highlighted the role of IBAK in regulating the biosynthesis of secondary metabolites pathway, potentially contributing to rice stress adaptability. The results of this study can provide new sustainable development solutions for agricultural production.

## 1 Introduction

Saline soils contain sufficient soluble salts to impair their productivity (Chhabra, 2004). Excessive salt accumulation in the root zone negatively affects plant growth (Pearson and Bauder, 2006). Due to the high salinity in the soil, the osmotic pressure increases, weakening plants’ water absorption capacity (dos Santos et al., 2022). When the osmotic pressure of plant cells is less than that of soil solution, plants encounter difficulty absorbing water. In addition, salt stress causes plants to absorb more sodium and chloride ions (Ketehouli et al., 2019). The intracellular ion homeostasis is destroyed, resulting in ion toxicity and mineral nutrient deficiency, affecting the normal growth of plants (Guo et al., 2020). In recent years, with the increasing population pressure, attention has been paid to developing and utilizing widely distributed saline-alkali wastelands to alleviate the food crisis.

Plant growth regulators are a class of substances that have similar physiological and biological effects to plant hormones. They have been widely used in field crops (Fahad et al., 2016; Amoanimaa-Dede et al., 2022), fruit trees (Bons and Kaur, 2020), and vegetables (Rahman et al., 2015) because of their significant and efficient regulating effects, which have played a particular role in promoting agricultural production. For example, diethyl aminoethyl hexanoate prolongs dormancy of storage organs, kinetin promotes seed germination (Araújo et al., 2019), paclobutrazol promotes rooting (Li et al., 2023), and gibberellin inhibits flower formation (Yamaguchi et al., 2014). Auxin is also a widely studied plant hormone that improves plant resistance to abiotic stress. Previous studies found that exogenous application with auxins such as indole-3-acetic acid (IAA) can effectively improve several crops’ salt stress resistance (Ashraf and Foolad, 2005; Ashraf, 2010; Kaya et al., 2013; Ribba et al., 2020).

Potassium indole-3-butyric acid (IBAK), whose chemical formula is C_12_H_12_KNO_2_, is a water-soluble plant growth regulator. It can act on vigorous growth parts, such as roots, buds, and fruits, and shows strong cell division and growth promotion on specific treatment sites. A recent study showed that spraying an IBAK solution at 80 mg L^−1^ on rice leaves at the jointing stage can regulate K^+^ and Na^+^ contents, increase net photosynthetic rate, increase catalase (CAT) activity and glutathione (GSH) content, and regulate the expression of genes related to carbohydrate metabolism under salt stress, indicating that IBAK played a role in enhancing the tolerance of rice to salt stress (Zhou et al., 2023). Zhou et al. (2023) preliminary revealed IBAK had a regulatory effect on rice carbohydrate metabolism. However, the changes in key enzymes and metabolites related to carbon metabolism still need to be further explored. In addition, because one of the functions of transcription factors is to improve plant stress resistance (Liu et al., 2023); secondary metabolites participate in protective functions in response to abiotic stress (Akula and Ravishankar, 2011). We speculated that IBAK may play a role in regulating TF gene expression and biosynthesis of secondary metabolites, which are also the focus of this study.

Rice is particularly sensitive to salt stress at the seedling stage (Moradi and Ismail, 2007). Based on previous research, this study decided to reveal the regulatory role of IBAK in carbon metabolism, TF genes expression, and biosynthesis of secondary metabolites under salt stress at the seedling stage through comprehensive transcriptome, metabolome, and physiological perspectives, filling the research gap and providing a new sustainable development solution for agricultural production.

## 2 Materials and methods

### 2.1 Experimental design

This study was conducted in the outdoor greenhouse of Guangdong Ocean University in 2021. The entire process was conducted under natural light, with day/night temperatures of 30/25 ± 2°C and 60% relative humidity. The rice variety used in this study was Xiangliangyou900. The concentration of the IBAK solution was 40 mg L^−1^, and distilled water was used as a control. First, the sterilized seeds were soaked in water for 24 h at room temperature. Subsequently, the seeds were primed for 24 h at room temperature. 57 germinated seeds were sown in flower pots of 19 cm × 14 cm × 17 cm containing 2.65 kg of brick-red soil. The physical and chemical properties of the soil were as follows: pH 7.23; available phosphorus, 4.05 mg kg^−1^; rapidly available potassium, 48.37 mg kg^−1^; alkali-hydrolyzale nitrogen, 37.10 mg kg^−1^; and organic matter, 32.44 mg kg^−1^. NaCl treatment was started on the eight day after sowing (4.82g NaCl was evenly integrated into the soil). The leaves were sprayed with IBAK solution 3 days after the salt treatment. Samples were collected 7 days after IBAK treatment. Except for the first leaf, all the remaining leaves were collected to detect carbohydrate content and enzyme activity related to carbon metabolism. The latest fully expanded leaves were collected for transcriptomic, metabolomic, and quantitative real-time PCR detection. There were three treatments in this study: CK0 (freshwater treatment), CK03 (salt treatment), and IBAK03 (IBAK, salt treatment). Three replicates were set for each treatment.

### 2.2 Determination of plant height, fresh weight, dry weight, leaf area, root shoot ratio, and moisture content

After harvest, the plant height, shoot fresh weight, shoot dry weight, root fresh weight, root dry weight, shoot moisture content, root moisture content, leaf area, and root shoot ratio were measured. Leaf area was obtained by YMJ-PC leaf area analysis system. Moisture content was calculated according to formula 1; root shoot ratio was calculated using formula 2.

Moisture content = (fresh weight − dry weight)/fresh weight (Formula 1)

Root shoot ratio = root dry weight/shoot dry weight (Formula 2)

### 2.3 Determination of carbohydrate content

The contents of soluble sugar, sucrose, fructose, and starch were determined according to the description of Meng et al. (2023).

Leaf samples (0.5 g) were ground into homogenate with an 80% ethanol solution (v/v). The mixture was then centrifuged at 4,000 rpm for 5 min at 80°C after 20 min in a water bath. The supernatant was collected and fixed to 25 mL to determine fructose, soluble sugar, and sucrose content. The sediment was used to measure starch content.

### 2.4 Determination of the activity of enzymes related to carbon metabolism

AI and NI activity were determined according to the description of Wang et al. (2024) and Meng et al. (2023).

### 2.5 Transcriptome sequencing

Shenzhen BGI Technology Co., Ltd., performed transcriptome detection work.

The secondary structure of the RNA sample was opened, and oligo(dT) magnetic beads were used to enrich the mRNA. A fragmentation reagent was added to the obtained mRNA to fragment the mRNA. The reaction system was prepared to synthesize one-strand cDNA and two-strand cDNA. The double-stranded cDNA ends were repaired, and an A base was added to the 3′ end. An adapter ligation reaction system was prepared to ligate adapters to cDNA. The PCR reaction system was prepared to amplify the product. The library was quality checked. After denaturing the PCR product into a single-stranded product, a cyclization reaction system was prepared. The single-stranded circular product was obtained, and the uncirculated linear DNA molecules were digested. Finally, sequencing was performed by combined probe-anchored polymerization technology (according to the method description provided by Shenzhen BGI Technology Co., Ltd.).

### 2.6 Metabolome detection and analysis

Shenzhen BGI Technology Co., Ltd., performed the metabolome detection work.

In this project, untargeted metabolomics analysis was performed using liquid chromatography with tandem mass spectrometry (LC-MS/MS), and high-resolution mass spectrometer Q Exactive (Thermo Fisher Scientific, USA) was used for data acquisition in positive-ion and negative-ion mode, respectively, to improve the metabolite coverage. LC-MS/MS data processing by Compound Discoverer 3.1 (Thermo Fisher Scientific, USA) mainly included peak extraction, peak alignment, and compound identification. The BGI’s own metabolomics software package metaX and metabolome information analysis process was used for data preprocessing, statistical analysis, metabolite classification, and functional annotation. The dimensionality of the original data of multiple variables was reduced by principle component analysis so as to analyze the grouping, trend (similarities and differences within and between sample groups), and outliers (whether there were abnormal samples) of the observed variables in the data set. The project used variable importance in projection (VIP) values of the first two principal components in multivariate partial least squares-discriminant analysis (PLS-DA) model, combined with fold change and Student’s t-test of univariate analysis to choose differentially expressed metabolites. Differential metabolites screening criteria: (1) the VIP values of the first two PCs of the PLS-DA model ≥1; (2) fold change ≥1.2 or ≤0.83; and (3) p-value <0.05.

### 2.7 Quantitative real-time PCR

Quantitative real-time PCR experiment was performed by Sangon Biotech (Shanghai) Co., Ltd. The primers used for quantitative real-time PCR analysis were listed in **Supplementary Table S1**.

## 3 Results

### 3.1 Effects of IBAK on morphology, fresh weight, dry weight, root shoot ratio, and moisture content of rice seedlings under salt stress

**Figure 1:**
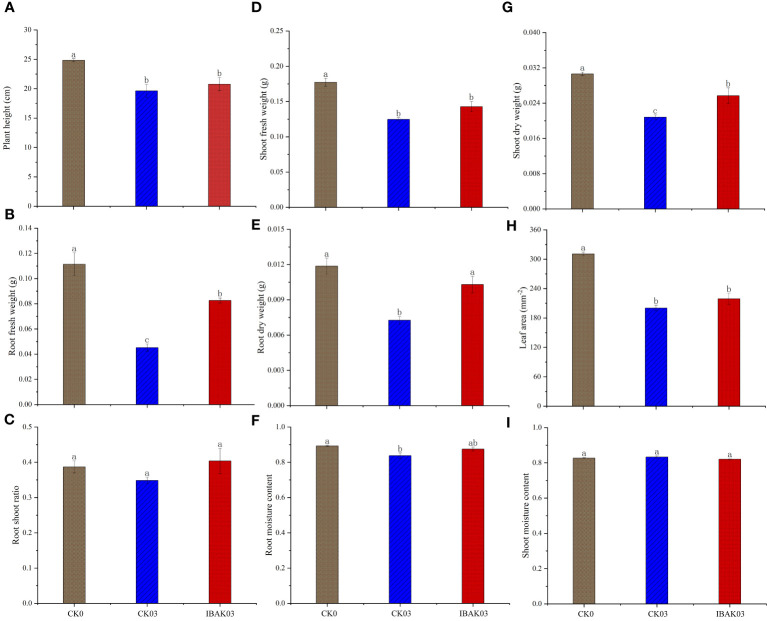
Effects of IBAK on morphology, fresh weight, dry weight, root shoot ratio, and moisture content of rice seedlings under salt stress. **(A)** plant height; **(B)** root fresh weight; **(C)** root shoot ratio; **(D)** shoot fresh weight; **(E)** root dry weight; **(F)** root moisture content; **(G)** shoot dry weight; **(H)** leaf area; **(I)** shoot moisture content.

As shown in **Figure 1**, salt stress significantly inhibited rice growth; compared with CK0, the plant height, fresh weight, dry weight, leaf area, and root moisture content of CK03 were significantly reduced. At the same time, this study found that the application of IBAK under salt stress had a certain alleviation effect; for example, the root fresh weight, root dry weight, and shoot dry weight of IBAK03 were significantly higher than those of CK03 by 82.81%, 41.68%, and 23.38%, respectively; other indicators were also improved to a certain extent but did not reach a significant level, such as shoot fresh weight, root shoot ratio, root moisture content, and leaf area.

**Figure 2:**
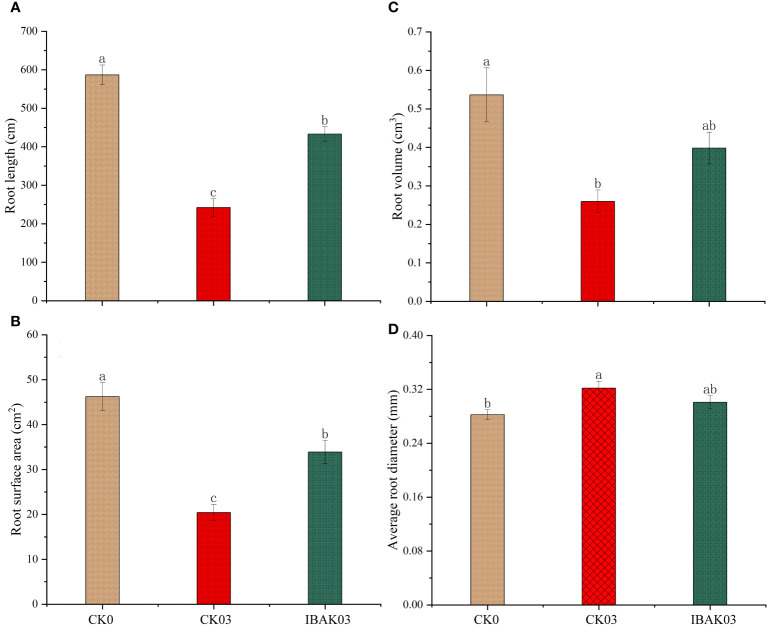
Effects of IBAK on root morphology under salt stress. **(A)** root length; **(B)** root surface area; **(C)** root volume; **(D)** average root diameter.

In addition, salt stress significantly reduced rice root length, root surface area, and root volume and significantly increased the average root diameter. Compared to CK03, spraying IBAK under salt stress significantly increased the root length and surface area by 78.97% and 65.99%, respectively, promoting rice root growth (**Figure 2**).

### 3.2 Effect of IBAK on carbohydrate content and carbon metabolism-related enzyme activity under salt stress

**Figure 3:**
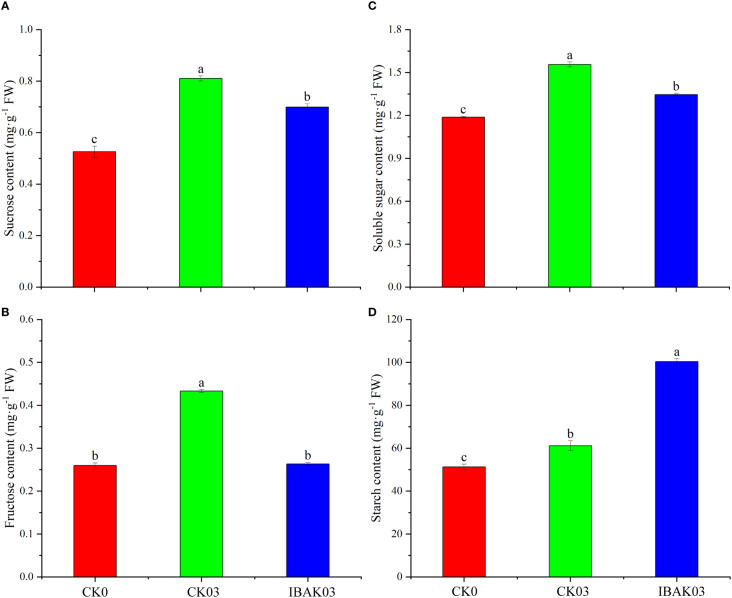
Effect of IBAK on sucrose, fructose, soluble sugar, and starch contents in rice leaves under salt stress. **(A)** sucrose content; **(B)** fructose content; **(C)** soluble sugar content; **(D)** starch content.

As shown in **Figure 3**, salt stress can significantly increase the sucrose, fructose, soluble sugar, and starch contents in rice leaves. Compared with CK03, the sucrose, fructose, and soluble sugar contents of IBAK03 were significantly reduced by 13.74%, 39.23%, and 13.51%, respectively; the starch content was significantly increased by 64.13%.

**Figure 4:**
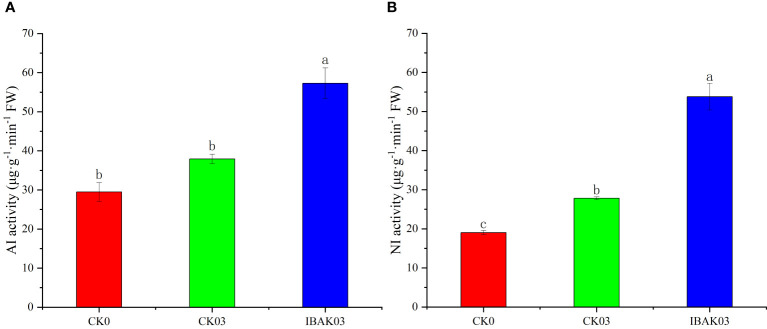
Effect of IBAK on AI and NI activity under salt stress. **(A)** acid invertase; **(B)** neutral invertase.

As shown in **Figure 4**, the NI activity of CK03 was significantly improved compared with CK0. The activities of AI and NI were significantly increased by 51.08% and 93.34% after using IBAK under salt stress compared with CK03.

### 3.3 Transcriptome

#### 3.3.1 Quality control

This study had nine samples, each producing an average of 6.62 Gb of data (**Supplementary Table S2**). The clean reads Q30 was greater than 89.63% (**Supplementary Table S2**). Reference genome alignment showed a total mapping rate of over 82.63% and a uniquely mapping rate of over 80.89% (**Supplementary Table S3**). Reference gene alignment showed a total mapping rate of over 70.13% and a uniquely mapping rate of over 66.21% (**Supplementary Table S4**). To verify the accuracy of the transcriptome data, quantitative real-time PCR was used to detect the expression levels of several randomly selected differentially expressed genes (DEGs). The results showed that expression patterns were similar to the transcriptome data, demonstrating the reliability of the RNA-seq data (**Supplementary Table S1**).

#### 3.3.2 DEGs statistics

**Figure 5:**
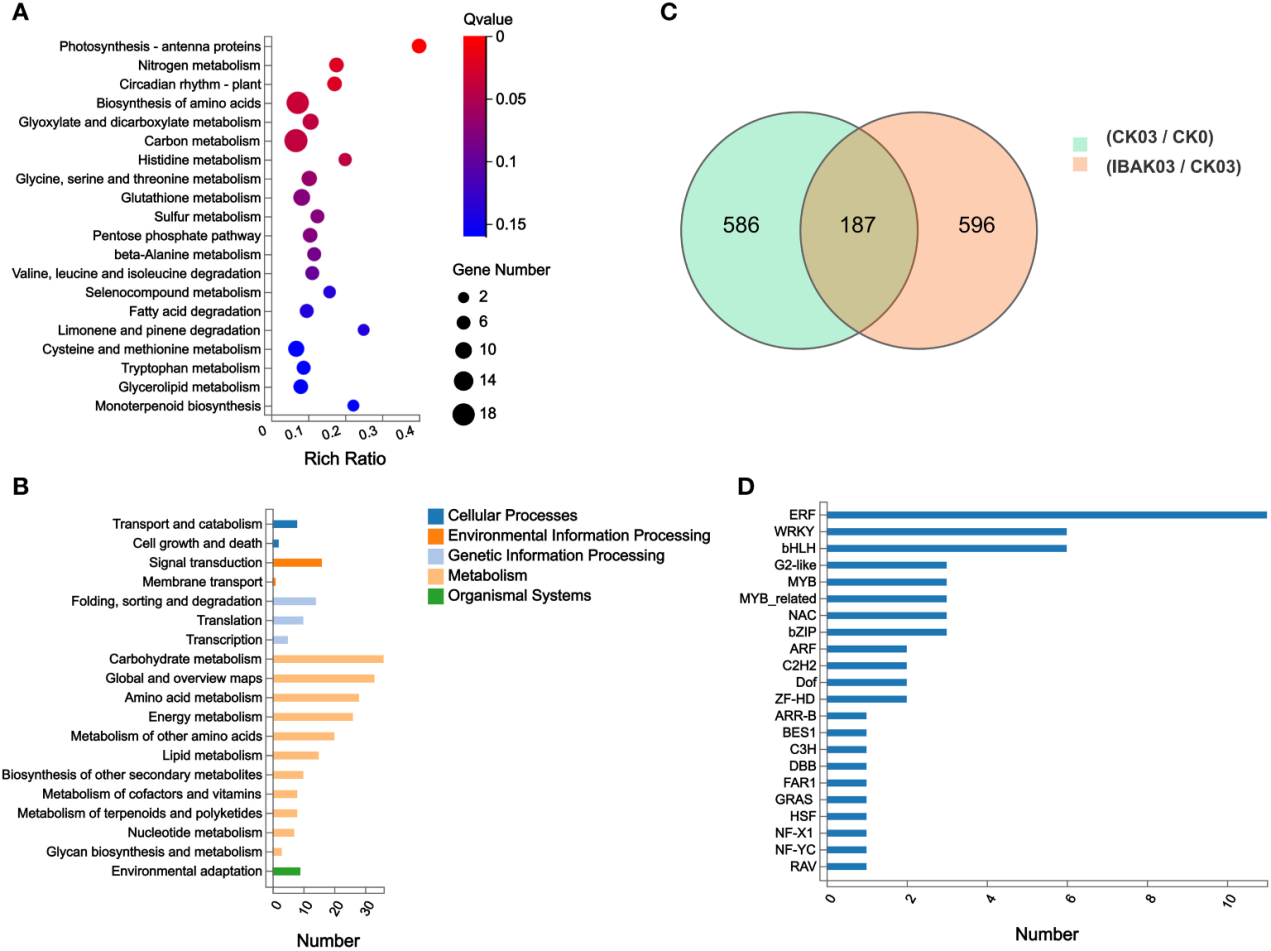
DEGs statistics, KEGG annotation analysis, KEGG enrichment analysis, and transcription factor genes statistics. **(A)** KEGG enrichment analysis; **(B)** KEGG annotation analysis; **(C)** DEGs statistics; **(D)** transcription factor genes statistics.

In this study, 773 DEGs were detected in the CK03/CK0 comparison group, of which 431 were upregulated and 342 were down-regulated. In the IBAK03/CK03 comparison group, 783 DEGs were detected, of which 300 were upregulated and 483 were down-regulated (**Figure 5C**; **Supplementary Tables S5**, **S6**).

#### 3.3.3 DEGs analysis

This study focused on analyzing 783 DEGs in the IBAK03/CK03 comparison group. Kyoto encyclopedia of genes and genomes (KEGG) annotation analysis found DEGs were mainly classified into transport and catabolism in Cellular Processes; signal transduction in Environmental Information Processing; folding, sorting and degradation in Genetic Information Processing; carbohydrate metabolism, global and overview maps, and amino acid metabolism in Metabolism; and environmental adaptation in Organismal Systems (**Figure 5B**).

**Figure 6:**
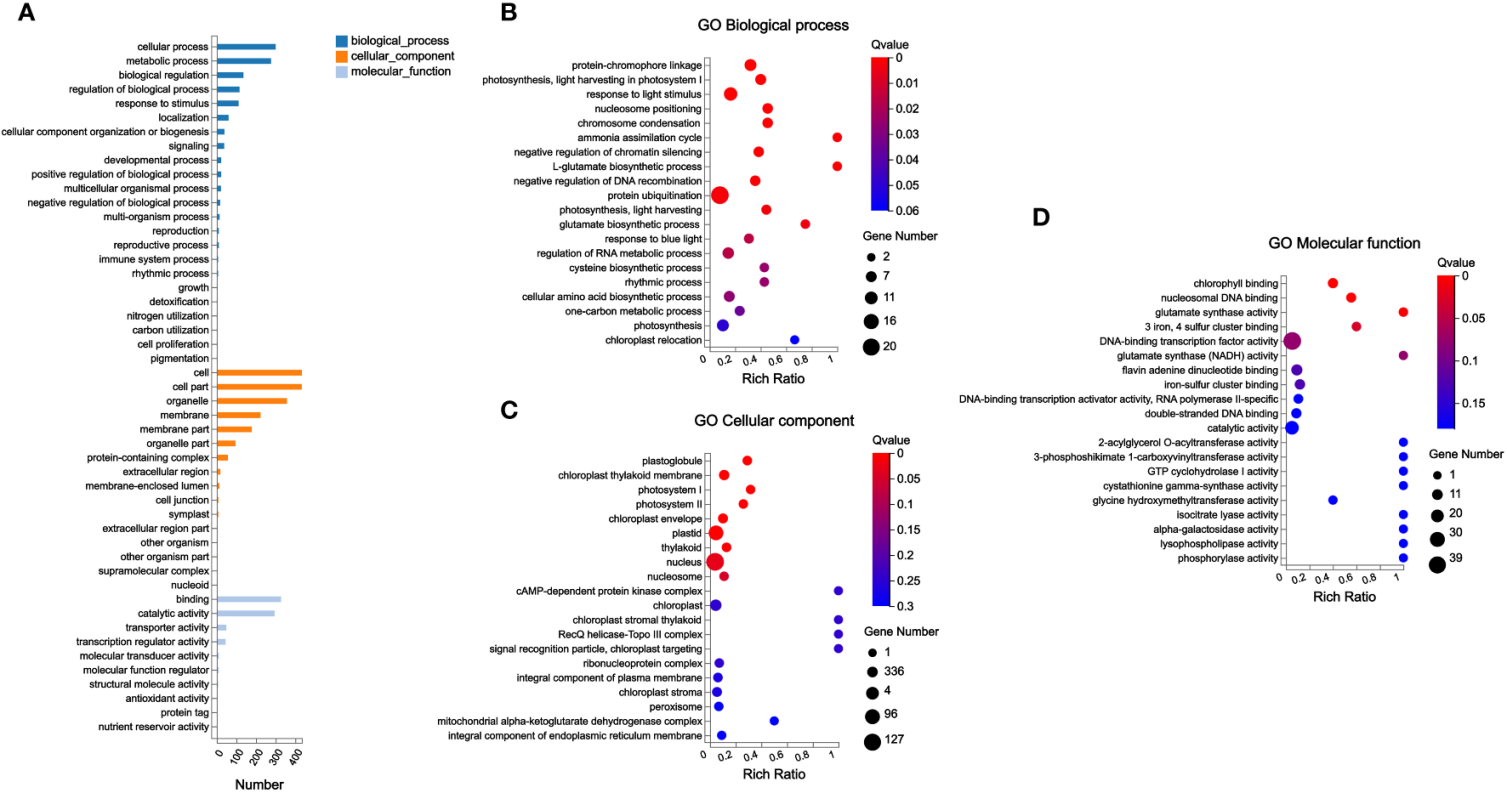
GO annotation analysis and GO enrichment analysis of DEGs. **(A)** GO annotation analysis; **(B–D)** GO enrichment analysis.

At the same time, this study conducted gene ontology (GO) annotation analysis on these 783 DEGs. The abundant DEGs were classified into cellular process and metabolic process in Biological Process; cell and cell part in Cellular Component; and binding and catalytic activity in Molecular Function (**Figure 6A**).

KEGG enrichment analysis was performed to understand these genes’ functions further. The results showed that photosynthesis - antenna proteins, nitrogen metabolism, circadian rhythm - plant, biosynthesis of amino acids, glyoxylate and dicarboxylate metabolism, carbon metabolism, and histidine metabolism were the most significant pathways enriched by DEGs; (**Figure 5A**).

In addition, GO enrichment analysis found that plastoglobule, chloroplast thylakoid membrane, photosystem I, photosystem II, chloroplast envelope, plastid, thylakoid, nucleus, and nucleosome were the significantly enriched terms in Cellular Component in this study; among these significantly enriched terms, the most abundant genes were enriched in nucleus. Chlorophyll binding, nucleosomal DNA binding, glutamate synthase activity, and “3 iron, 4 sulfur cluster binding” were significantly enriched GO terms in Molecular Function. In Biological Process, a total of 19 GO terms were significantly enriched; among these significantly enriched GO terms, the most abundant gene was classified into protein ubiquitination (**Figures 6B–D**).

Finally, transcription factor classification was performed on the 783 DEGs detected in the IBAK03/CK03 comparison group. This study detected a total of 56 TF genes, and the abundant TF genes were classified into ERF, WRKY, and bHLH families (**Figure 5D**).

### 3.4 Metabolome

#### 3.4.1 Principal component analysis

**Figure 7:**
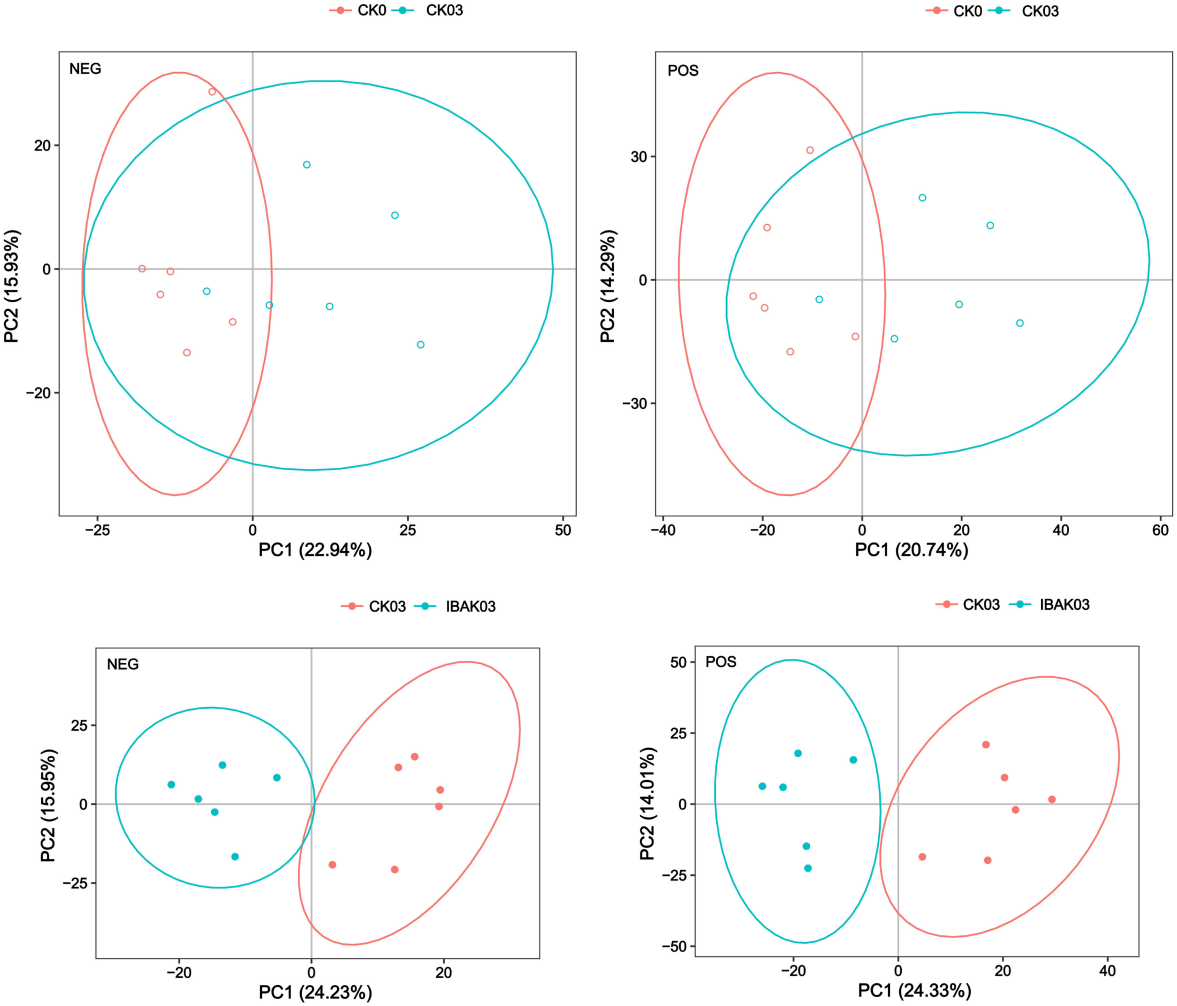
Principal component analysis.

The principal component analysis showed that the six samples of each treatment were within the 95% confidence interval. The six samples of CK0 were basically distinguished from those of CK03; the six samples of CK03 were completely separated from those of IBAK03, indicating differences between the two treatments (**Figure 7**).

#### 3.4.2 Partial least squares-discriminant analysis

PLS-DA is a statistical method for supervised discriminant analysis that best reflects the differences between taxonomic groups. The PLS-DA results showed significant differences between CK0 and CK03; similarly, CK03 and IBAK03 were completely separated. To judge the quality of the model, 200 response permutation testing were performed on the model of PLS-DA. In the positive or negative ion mode, the Q^2^ of the two comparison groups were all less than 0, indicating no overfitting phenomenon, and the model was reliable (**Supplementary Figures S1**, **S2**; **Supplementary Table S7**).

#### 3.4.3 Statistics and analysis of differential metabolites

In the positive ion mode, a total of 60 differential metabolites were detected in the CK03/CK0 comparison group, of which 30 were upregulated and 30 were down-regulated; in the negative ion mode, 34 differential metabolites were detected, of which 14 were upregulated, and 20 were down-regulated. For the IBAK03/CK03 comparison group, 120 differential metabolites were detected in the positive ion mode, of which 81 were upregulated, and 39 were down-regulated; 60 differential metabolites were detected in the negative ion mode, of which 31 were upregulated, and 29 were down-regulated (**Supplementary Figure S3**; **Supplementary Table S8**).

In order to further understand the functional properties of metabolites, this study conducted KEGG functional annotation on the differential metabolites identified in the IBAK03/CK03 comparison group. In negative ion mode, most differential metabolites were classified into global and overview maps, amino acid metabolism, biosynthesis of other secondary metabolites, metabolism of cofactors and vitamins, and carbohydrate metabolism in Metabolism. In positive ion mode, the abundant differential metabolites were classified into global and overview maps, lipid metabolism, amino acid metabolism, and biosynthesis of other secondary metabolites in Metabolism (**Supplementary Figure S4A**).

In addition, KEGG enrichment analysis found a total of 31 significantly enriched KEGG pathways were found in negative ion mode in the IBAK03/CK03 comparison group; the differential metabolites were mainly enriched in phenylalanine metabolism, citrate cycle (TCA cycle), tyrosine metabolism, “alanine, aspartate and glutamate metabolism,” butanoate metabolism, biosynthesis of secondary metabolites, phenylpropanoid biosynthesis, and carbon metabolism pathway. In the positive ion mode, a total of 5 KEGG pathways were significantly enriched, namely, phenylpropanoid biosynthesis, linoleic acid metabolism, phenylalanine metabolism, biosynthesis of secondary metabolites, and sphingolipid metabolism (**Supplementary Figure S4B**).

## 4 Discussion

IBAK is the potassium salt form of indole-3-butyric acid (IBA). IBA occurs naturally in many plants (Blommaert, 1954; Bayer, 1969; Sutter and Cohen, 1992). A previous study found that IBA can increase the number of lateral roots in the seminal root in rice, but IAA cannot, and the study pointed out that the signal transduction pathway for IBA was at least partially different from that for IAA (Wang et al., 2003). The auxin action of IBA has been suggested to be due to its conversion to IAA (Chhun et al., 2004). However, a study found that the stimulatory effect of IBA on lateral root development was not through its conversion to IAA in rice (Chhun et al., 2004). These studies indicated that there are differences between the response mechanisms of rice to IAA and IBA. IBA was reported to play a positive role in alleviating abiotic stress in plants (Tammam, 2009; Li et al., 2018). However, compared with IBA, the use of IBAK introduces potassium ions. Therefore, the action mechanism of IBAK on plants may be more complex. The results of this study showed that the root dry and fresh weight, root length, and root surface area were significantly increased after applying IBAK under salt stress, proving that IBAK had the function of promoting root growth. These results are similar to the findings by Jemaa et al. (2011) in *Arabidopsis*; their study found that IBA still retained the ability to stimulate lateral root growth under salt stress. Furthermore, the dry matter weight of the shoot in IBAK03 was higher than CK03. These results indicated that IBAK promoted rice growth under salt stress.

### 4.1 IBAK regulated carbon metabolism under salt stress

Carbon metabolism refers to the process in which plants convert carbon dioxide into organic substances (Dusenge et al., 2019), and it is the foundation of plant life activities. Zhou et al. (2023) found that spraying IBAK on rice leaves at the jointing stage can significantly upregulate two starch synthase genes: *OsSSIIb* (LOC4330709) and *OsGBSSII* (LOC4343010). The difference is that no significant changes were found in the expression levels of these two genes in the IBAK03/CK03 comparison group in this study. We speculated that this difference may be due to the different responses of rice at various growth stages to external stimuli or that different concentrations of IBAK solution may lead to different biological effects. Starch content was increased in IBAK03 compared with CK03. This may be related to another significantly upregulated starch synthase gene *OsSSIIc* (LOC4348711) detected in this study. In addition, Zhou et al. (2023) reported that IBAK treatment upregulated the gene *OsUgp1* (LOC4347800), which is a UDP-glucose pyrophosphorylase gene, and its upregulation may contribute to the synthesis of sucrose. However, the expression level of this gene did not change significantly in this study. Meanwhile, this study found that sucrose, fructose, and soluble sugar were significantly reduced in IBAK03 compared with CK03. Therefore, we speculated that using IBAK (40 mg L^−1^) under salt stress may adjust rice carbon allocation and improve starch storage at the seedling stage. Rice may use more carbon for starch synthesis instead of sucrose and fructose synthesis. In addition, the activities of AI and NI increased after using IBAK under salt stress. The increase in the activities of these enzymes may be one of the reasons for the decrease in sucrose content in this study. These two enzymes are involved in decomposing sucrose into fructose and glucose. Increasing AI and NI activity can regulate rice energy supply under salt stress by accelerating sucrose decomposition.

KEGG enrichment analysis on DEGs in the IBAK03/CK03 comparison group found that spraying IBAK upregulated the gene *OsSHM1* (LOC4334048) enriched in the carbon metabolism pathway. Wang et al. (2015) observed the growth status of *osshm1* mutant seeds in the greenhouse and found that the newly grown leaves after seed germination showed a decrease in the contents of total chlorophyll, chlorophyll a, chlorophyll b, and carotenoids; *osshm1* produced higher ROS and increased O_2_^−^ and H_2_O_2_ accumulation. Using IBAK to upregulate *OsSHM1* may be beneficial in reducing oxidative stress damage to rice under salt stress. In addition, gene *OsPPDKB* (LOC4338750) was upregulated in the IBAK03/CK03 comparison group. This gene is an essential regulator of carbon flux during starch and fat biosynthesis during rice filling (Kang et al., 2005).

In addition, spraying IBAK upregulated 2-oxoglutaric acid, fumaric acid, and succinic acid in the carbon metabolism pathway under salt stress (**Supplementary Table S9**). 2-oxoglutarate is a crucial metabolite at the crossroads of carbon/nitrogen metabolism as it is required for ammonia assimilation (Hodges, 2002; Araújo et al., 2014). Succinic acid has already been shown to be a stimulant of plant respiration (Bennet-Clark and Bexon, 1943; Turner and Hanly, 1949). In TCA cycle, 2-oxoglutaric acid is located after isocitrate and before succinyl coenzyme A. Succinic acid undergoes a series of reactions to finally generate oxaloacetate. The upregulation of these metabolites may increase the activity of the TCA cycle.

### 4.2 IBAK regulated biosynthesis of secondary metabolites pathway under salt stress

Secondary metabolites participate in protective functions in response to abiotic stress conditions (Akula and Ravishankar, 2011). In this study, many differential metabolites were enriched in the biosynthesis of secondary metabolites pathway in the IBAK03/CK03 comparison group. Specifically, this study found that 7-methylxanthine, 2-oxoglutaric acid, fumaric acid, succinic acid, L-phenylalanine, AICA ribonucleotide, 2-hydroxycinnamic acid, cinnamic acid, and 12-oxo phytodienoic acid were upregulated after using IBAK under salt stress (**Supplementary Table S9**, **S10**). 2-oxoglutarate (2-OG) is a crucial organic acid of the TCA (Lancien et al., 2000; Scheible et al., 2000; Araújo et al., 2014). It is also an obligatory substrate in a range of oxidative reactions catalyzed by 2-OG–dependent dioxygenases (Araújo et al., 2014). Fumaric acid is another essential component of the TCA cycle and can be metabolized to produce energy and carbon skeletons for the production of other compounds (Chia et al., 2000). 12-oxo-phytodienoic acid is the major precursor of (-)-jasmonic acid, playing a role in activating and fine-tuning defense responses, as well as plant growth processes (Liu and Park, 2021). These changes may reflect the impact of IBAK on the metabolic regulation and defense mechanisms of plants under salt stress.

### 4.3 IBAK regulated TF gene expression under salt stress

In this study, abundant TF genes were classified into ERF, WRKY, and bHLH families in the IBAK03/CK03 comparison group. These transcription factor families play essential roles in plant responses to abiotic stress. This study found that 11 TF genes belong to the ERF family. According to reports, several ERFs have been shown to be involved in plant stress-response processes, such as salt and drought (Zhang et al., 2009; Zhang and Huang, 2010; Yang et al., 2011; Cheng et al., 2019; Li et al., 2019; Huang et al., 2020). The bHLH family is also one of the largest transcription factor families in plants and plays an essential role in plant response to salt stress (Qian et al., 2021). LOC4345984 (transcription factor bHLH130), belonging to the bHLH family, was upregulated in this study. Previous studies reported that MdbHLH130 acts as a positive regulator of water stress response by regulating stomatal closure and reactive oxygen species (ROS)-scavenging in tobacco (Zhao et al., 2020; Qian et al., 2021). These results provided new clues for further research on the mechanism of IBAK regulating rice stress resistance by affecting TF gene expression.

## 5 Conclusion

The changes in multiple indicators in this study proved that IBAK can promote rice growth under salt stress. This study indicated that IBAK can change carbon allocation, increase starch reserves, and promote sucrose decomposition. Meanwhile, IBAK upregulated many key metabolites related to plant stress response and growth regulation in the biosynthesis of secondary metabolites pathway. In addition, multiple TF genes were differentially expressed, especially those in the ERF, WRKY, and bHLH families, which provided new clues for the mechanism of IBAK regulating rice resistance to salt stress.

## Data Availability

Transcriptome and metabolome raw data have been uploaded to online repositories. Transcriptome raw data was deposited in the Genome Sequence Archive in National Genomics Data Center, China National Center for Bioinformation / Beijing Institute of Genomics, Chinese Academy of Sciences that are publicly accessible at https://ngdc.cncb.ac.cn/gsa accession number GSA: CRA018370. Metabolome raw data was deposited in the OMIX, China National Center for Bioinformation / Beijing Institute of Genomics, Chinese Academy of Sciences: https://ngdc.cncb.ac.cn/omix with accession no. OMIX007146. 1. The Genome Sequence Archive Family: Toward Explosive Data Growth and Diverse Data Types. Genomics, Proteomics & Bioinformatics 2021, 19(4):578-583. https://doi.org/10.1016/j.gpb.2021.08.001 [PMID=34400360] 2. Database Resources of the National Genomics Data Center, China National Center for Bioinformation in 2024. Nucleic Acids Res 2024, 52(D1):D18-D32. https://doi.org/10.1093/nar/gkad1078 [PMID=38018256] 3 Database Resources of the National Genomics Data Center, China National Center for Bioinformation in 2022. Nucleic Acids Res 2022, 50(D1):D27-D38. https://doi.org/10.1093/nar/gkab951 [PMID=34718731].
