# Supplementary material for: Potassium indole-3-butyric acid affects rice’s adaptability to salt stress by regulating carbon metabolism, transcription factor genes expression, and biosynthesis of secondary metabolites
Source: Front Plant Sci. 2024 Sep 3;15:1416936. doi: 10.3389/fpls.2024.1416936 (PMC11405336; doi:10.3389/fpls.2024.1416936)
Supplement: Supplementary file 1 [file DataSheet1.zip › Supplementary materials/Supplementary Tables.docx]

Table.S1 Validation of selected genes using qRT-PCR

| ID | Primer sequence | Treatment | FPKM | Relative expression level |
| --- | --- | --- | --- | --- |
| 4346803 | F1 5' GCGTCCTCTACCTCGGCC 3' | CK03 | 747.42 | 0.922920744 |
|  | R1 5' TGTCCCACCCGTAGTCGC 3' | IBAK03 | 221.12 | 0.342783385 |
| 4328623 | F1 5' CGAGGCGGAGATGAAGGA 3' | CK03 | 1060.90 | 1.081481231 |
|  | R1 5' GGCTGGTGAGGATGTTGTTGT 3' | IBAK03 | 532.33 | 0.93632152 |
| 4333359 | F2 5' GGTGAAGTTCGGGGAGGC 3' | CK03 | 398.43 | 1.050900863 |
|  | R2 5' GAATCCCATGAGCACCACCT 3' | IBAK03 | 172.63 | 0.942238924 |

Table.S2 Filtered reads quality statistics

| Sample | Total raw reads (M) | Total clean reads (M) | Total clean bases (Gb) | Clean reads Q20 (%) | Clean reads Q30 (%) | Clean reads ratio (%) |
| --- | --- | --- | --- | --- | --- | --- |
| CK0 | 49.1 | 43.45 | 6.52 | 95.46 | 89.63 | 88.49 |
| CK0 | 45.82 | 40.1 | 6.02 | 95.67 | 90.03 | 87.51 |
| CK0 | 50.69 | 44.7 | 6.71 | 95.68 | 90.05 | 88.19 |
| CK03 | 50.83 | 44.93 | 6.74 | 95.75 | 90.11 | 88.38 |
| CK03 | 50.83 | 45.67 | 6.85 | 95.74 | 90.12 | 89.84 |
| CK03 | 52.59 | 44.42 | 6.66 | 95.7 | 90.11 | 84.46 |
| IBAK03 | 54.34 | 45.89 | 6.88 | 95.75 | 90.23 | 84.46 |
| IBAK03 | 50.83 | 44.37 | 6.66 | 95.83 | 90.33 | 87.29 |
| IBAK03 | 50.05 | 43.81 | 6.57 | 95.7 | 90.1 | 87.52 |

Table.S3 Reference genome alignment

| Sample | Total clean reads (M) | Total mapping(%) | Uniquely mapping(%) |
| --- | --- | --- | --- |
| CK0 | 43.45 | 84.7 | 82.89 |
| CK0 | 40.1 | 84.81 | 83.03 |
| CK0 | 44.7 | 85.19 | 83.36 |
| CK03 | 44.93 | 85.05 | 83.34 |
| CK03 | 45.67 | 85.34 | 83.47 |
| CK03 | 44.42 | 83.66 | 81.92 |
| IBAK03 | 45.89 | 82.63 | 80.89 |
| IBAK03 | 44.37 | 84.84 | 83 |
| IBAK03 | 43.81 | 84.62 | 82.76 |

Table.S4 Reference gene alignment

| Sample | Total clean reads (M) | Total mapping(%) | Uniquely mapping(%) |
| --- | --- | --- | --- |
| CK0 | 43.45 | 75.31 | 71.17 |
| CK0 | 40.1 | 73.82 | 69.79 |
| CK0 | 44.7 | 74.65 | 70.56 |
| CK03 | 44.93 | 73.17 | 69.53 |
| CK03 | 45.67 | 73.45 | 69.5 |
| CK03 | 44.42 | 71.91 | 68.18 |
| IBAK03 | 45.89 | 70.13 | 66.21 |
| IBAK03 | 44.37 | 73.91 | 69.74 |
| IBAK03 | 43.81 | 74.46 | 70.41 |

Table.S7 PLS-DA model parameters

| Mode | Group | A | R^2^Y(cum) | Q^2^(cum) | R^2^ | Q^2^ |
| --- | --- | --- | --- | --- | --- | --- |
| pos | CK03/CK0 | 3 | 1 | 0.63 | (0.0, 0.99) | (0.0, -0.62) |
| pos | IBAK03/CK03 | 3 | 1 | 0.77 | (0.0, 0.99) | (0.0, -0.72) |
|  |  |  |  |  |  |  |
| neg | CK03/CK0 | 3 | 1 | 0.57 | (0.0, 0.99) | (0.0, -0.69) |
| neg | IBAK03/CK03 | 3 | 1 | 0.81 | (0.0, 0.99) | (0.0, -0.70) |

A: the number of PCs. R^2^Y(cum): the interpretation rate of Y matrix. Q^2^(cum): the predictive capability; R^2^ and Q^2^ are the Y-axis intercepts of the R^2^ and Q^2^ regression lines during the response sequencing test.

Table.S8 Statistics of differential metabolites

| Mode | Group | Total number of differential metabolites | Up | Down |
| --- | --- | --- | --- | --- |
| Pos | CK03/CK0 | 60 | 30 | 30 |
| Pos | IBAK03/CK03 | 120 | 81 | 39 |
| Neg | CK03/CK0 | 34 | 14 | 20 |
| Neg | IBAK03/CK03 | 60 | 31 | 29 |
