# Supplementary figures and images for: Potassium indole-3-butyric acid affects rice’s adaptability to salt stress by regulating carbon metabolism, transcription factor genes expression, and biosynthesis of secondary metabolites
Source: Front Plant Sci. 2024 Sep 3;15:1416936. doi: 10.3389/fpls.2024.1416936 (PMC11405336; doi:10.3389/fpls.2024.1416936)

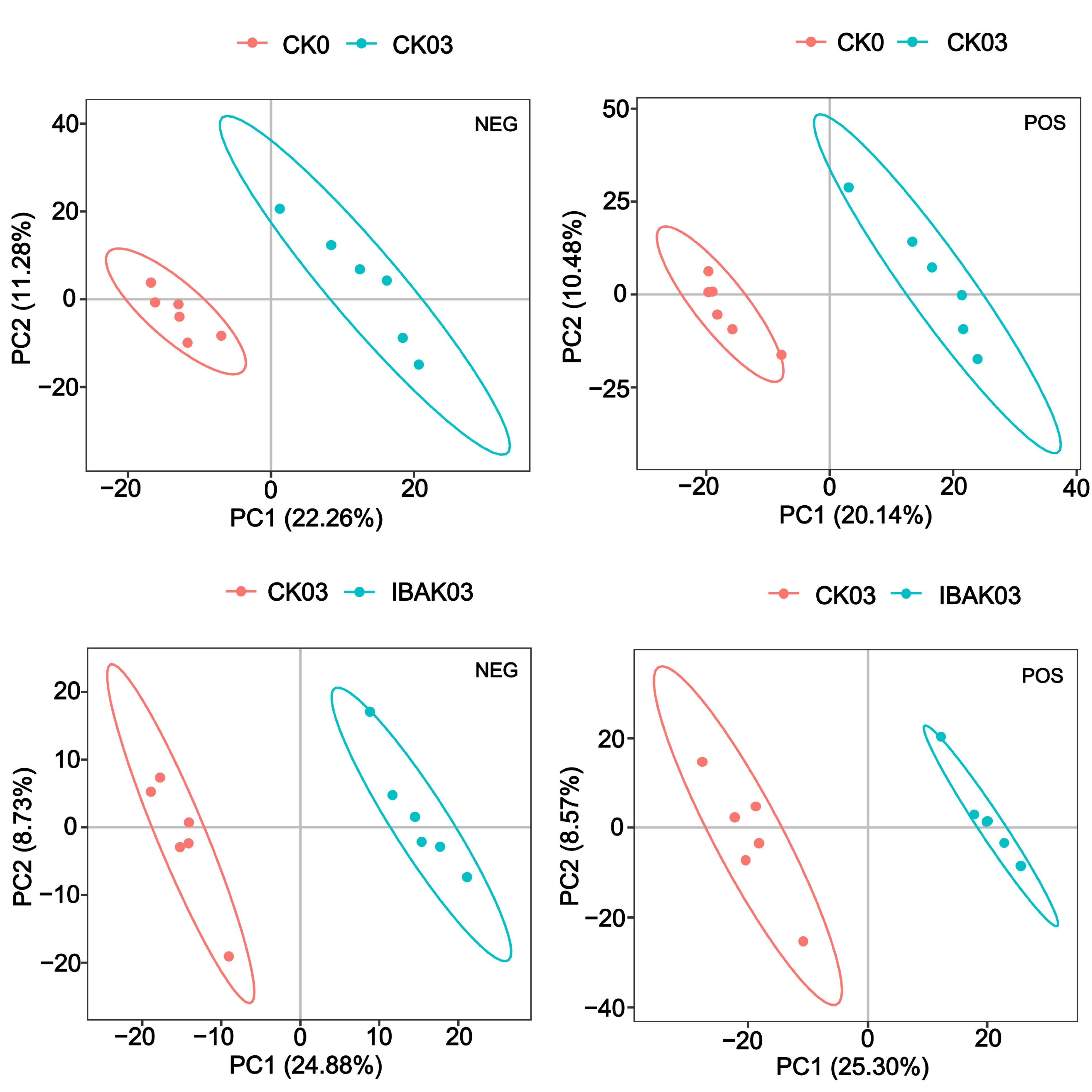

Supplement: Supplementary file 1 [file DataSheet1.zip › Supplementary materials/Supplementary Figure.S1 PLS-DA model score map.tif]

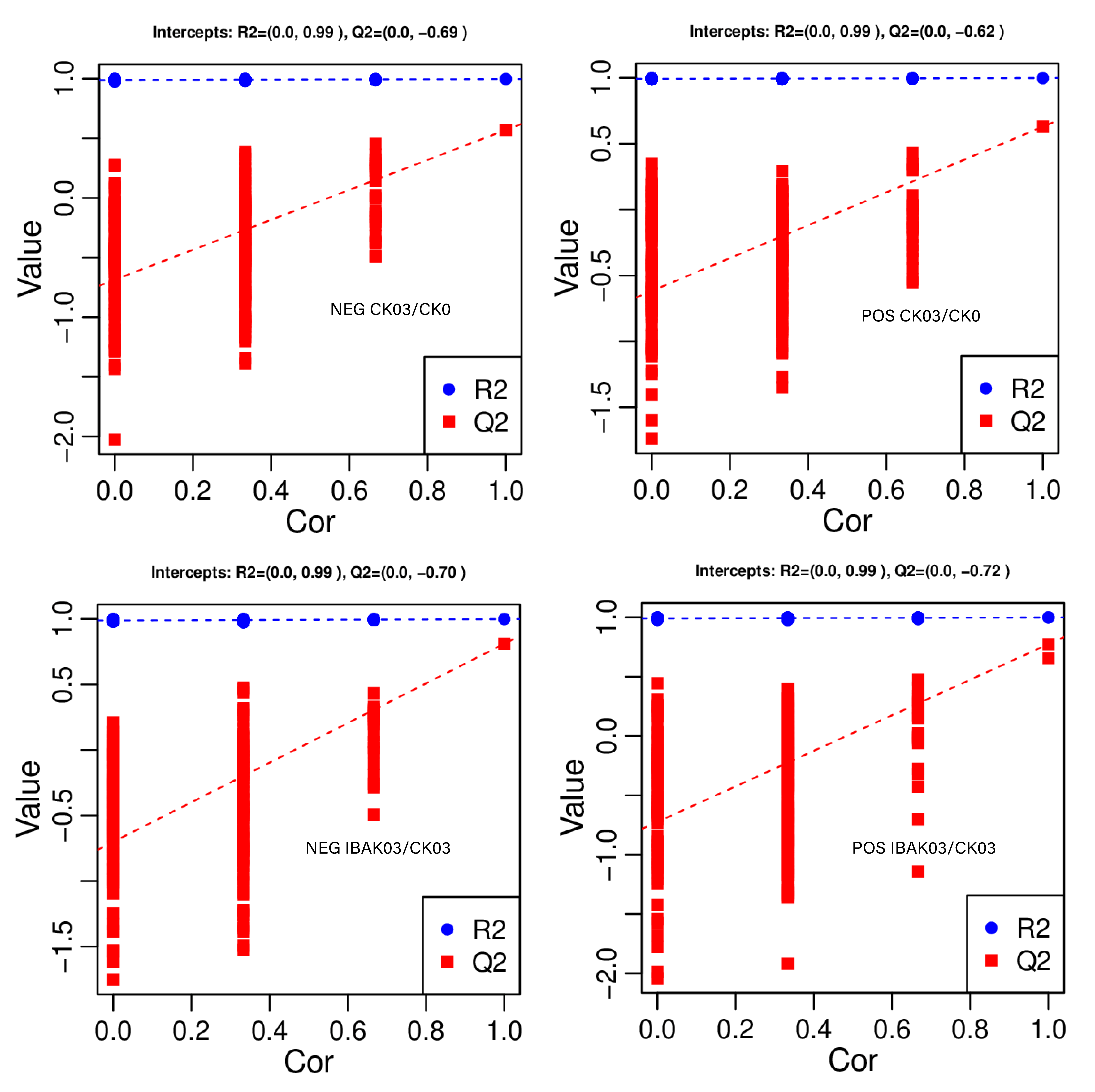

Supplement: Supplementary file 1 [file DataSheet1.zip › Supplementary materials/Supplementary Figure.S2 Response sequencing verification diagram of the PLS-DA model.tif]

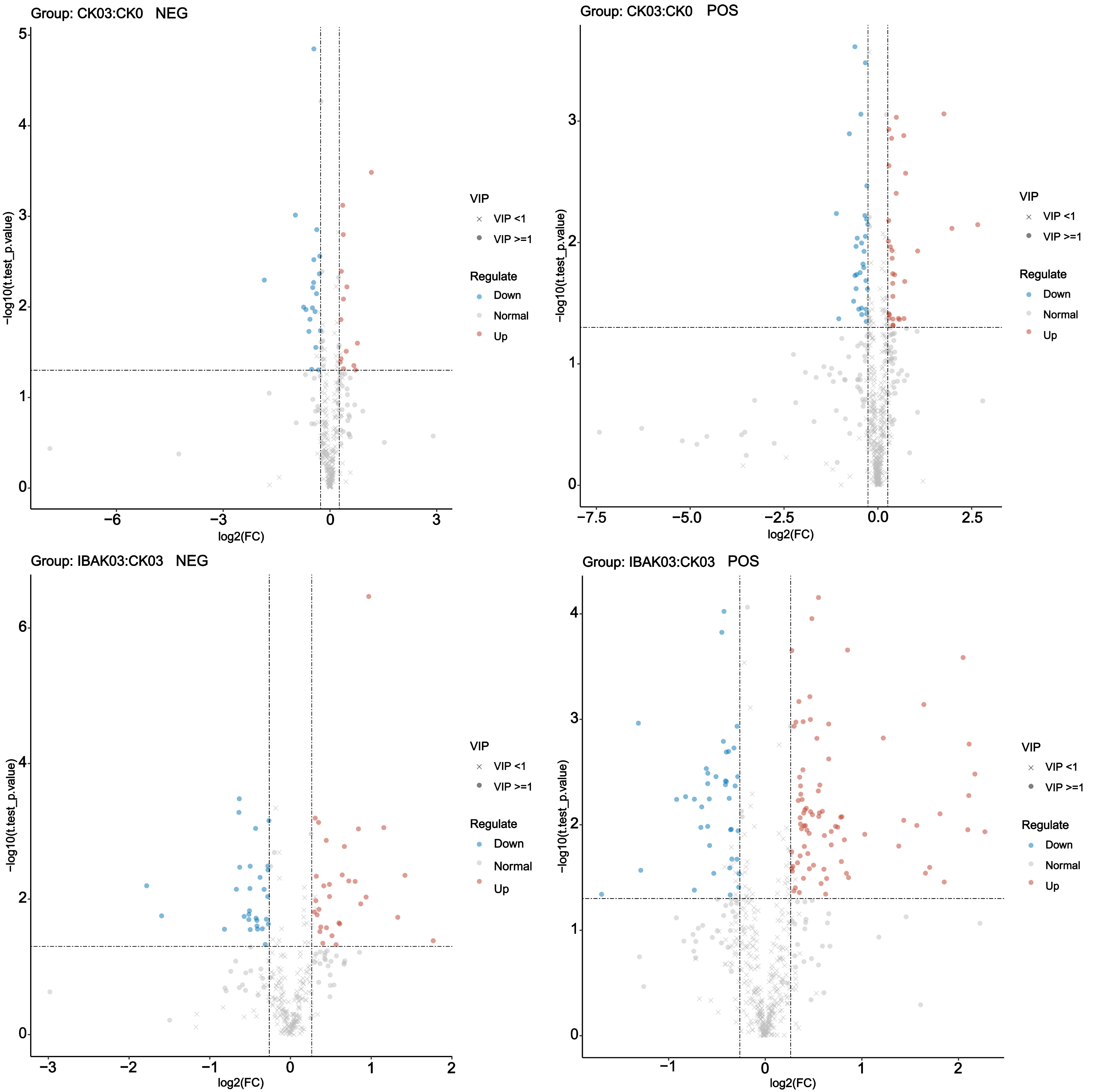

Supplement: Supplementary file 1 [file DataSheet1.zip › Supplementary materials/Supplementary Figure.S3 Differential metabolites volcano plot.tif]

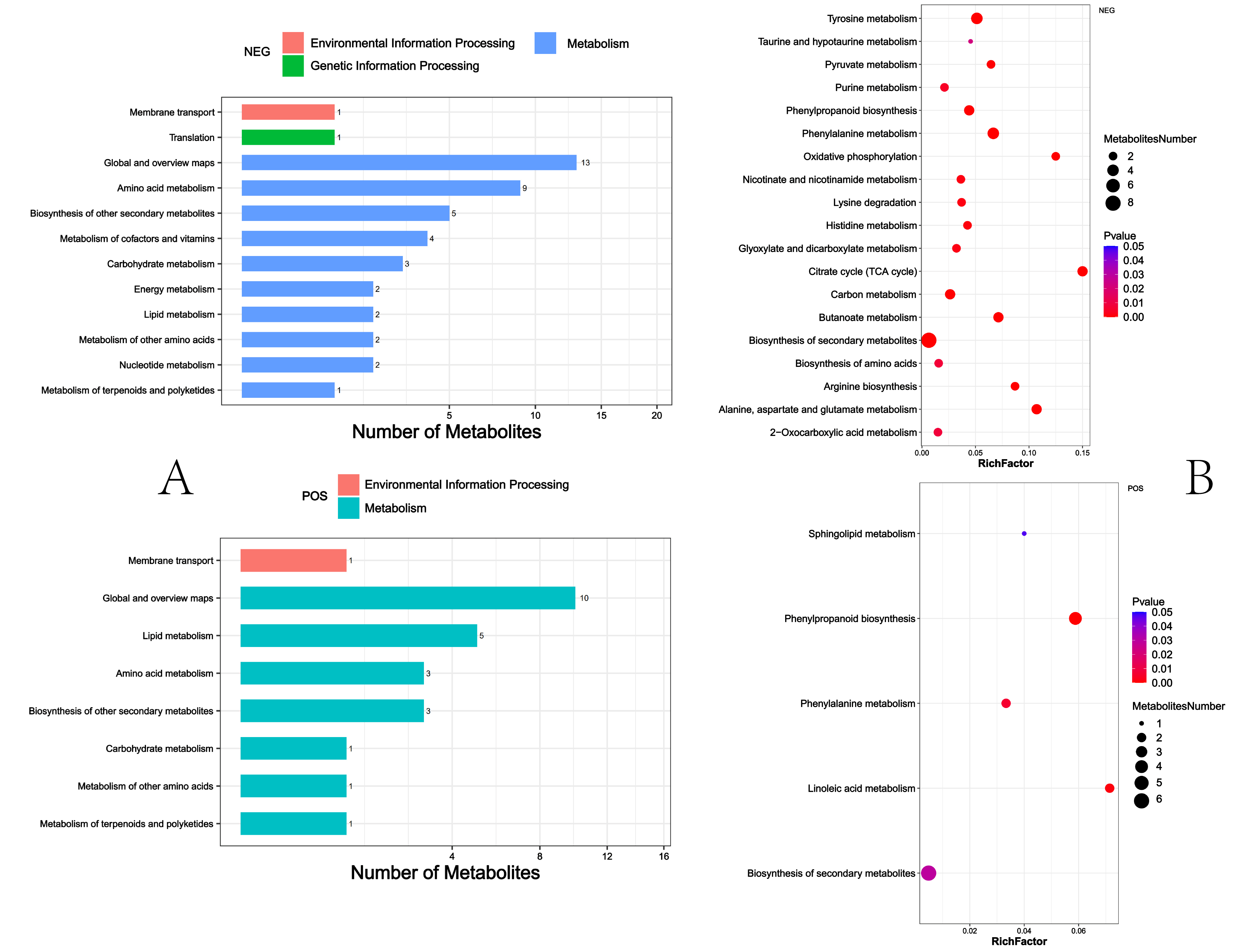

Supplement: Supplementary file 1 [file DataSheet1.zip › Supplementary materials/Supplementary Figure.S4 KEGG annotation analysis and KEGG enrichment analysis of differential metabolites.tif]
